# Modeling of Magnetic Films: A Scientific Perspective

**DOI:** 10.3390/ma17061436

**Published:** 2024-03-21

**Authors:** Denis Misiurev, Vladimír Holcman

**Affiliations:** Department of Physics, Faculty of Electrical Engineering and Communication, Brno University of Technology, Technicka 2848/8, 61600 Brno, Czech Republic; holcman@vut.cz

**Keywords:** magnetic films, substrates, first-principles calculation, density functional theory, electronic structure, molecular dynamics simulations, interface phenomena, Monte Carlo simulation

## Abstract

Magnetic thin-film modeling stands as a dynamic nexus of scientific inquiry and technological advancement, poised at the vanguard of materials science exploration. Leveraging a diverse suite of computational methodologies, including Monte Carlo simulations and molecular dynamics, researchers meticulously dissect the intricate interplay governing magnetism and thin-film growth across heterogeneous substrates. Recent strides, notably in multiscale modeling and machine learning paradigms, have engendered a paradigm shift in predictive capabilities, facilitating a nuanced understanding of thin-film dynamics spanning disparate spatiotemporal regimes. This interdisciplinary synergy, complemented by avantgarde experimental modalities such as in situ microscopy, promises a tapestry of transformative advancements in magnetic materials with far-reaching implications across multifaceted domains including magnetic data storage, spintronics, and magnetic sensing technologies. The confluence of computational modeling and experimental validation heralds a new era of scientific rigor, affording unparalleled insights into the real-time dynamics of magnetic films and bolstering the fidelity of predictive models. As researchers chart an ambitiously uncharted trajectory, the burgeoning realm of magnetic thin-film modeling burgeons with promise, poised to unlock novel paradigms in materials science and engineering. Through this intricate nexus of theoretical elucidation and empirical validation, magnetic thin-film modeling heralds a future replete with innovation, catalyzing a renaissance in technological possibilities across diverse industrial landscapes.

## 1. Introduction

In the realm of materials science and condensed matter physics, the intricate interplay between magnetic films and diverse substrates has emerged as a captivating avenue of research with far-reaching implications. The ability to tailor magnetic properties at the nanoscale has opened up a myriad of possibilities for technological advancements spanning from data storage and sensing to spintronics and quantum computing. Central to these developments is the indispensable role of modeling techniques, which empower researchers to decipher the complex dynamics and behavior of magnetic films on varied substrates.

Modeling techniques have revolutionized our comprehension of magnetic phenomena by providing invaluable insights into the underlying physical mechanisms, interactions, and responses. These techniques encompass a diverse array of methodologies, ranging from atomistic simulations to continuum approaches, each offering unique advantages and perspectives. As we delve into the scientific dimensions of modeling magnetic films on diverse substrates, it becomes evident that a comprehensive exploration of these techniques is essential to unraveling the intricate tapestry of magnetic behavior.

The primary focus of this review is to provide a comprehensive overview of the current state-of-the-art modeling techniques employed in studying magnetic films on diverse substrates. We aim to elucidate the underlying principles, capabilities, and limitations of these methods while shedding light on the varied perspectives they bring to the forefront. By synthesizing the collective knowledge amassed from both experimental observations and theoretical endeavors, we endeavor to contribute to a deeper understanding of the intricate interplay between magnetic films and substrates.

This exploration will be structured as follows: First, we will delve into the foundational concepts that underpin the magnetic interactions and phenomena observed in thin films on different substrates. Subsequently, we will embark on a journey through the realm of modeling techniques, beginning with atomistic simulations that capture the fundamental atomic-scale interactions. Moving forward, continuum models will take center stage, offering a broader perspective on the collective behavior of magnetic domains and their response to external stimuli.

As we navigate through these modeling techniques, we will highlight the unique insights they provide into various aspects of magnetic film–substrate systems, such as surface effects, domain wall dynamics, and magnetic anisotropy. Moreover, we will emphasize the importance of synergy between experimental observations and theoretical predictions, illustrating how modeling enriches our understanding by elucidating complex phenomena that may not be readily discernible through experiments alone.

## 2. Growth Mechanisms of Thin Films

The growth mechanism of thin films is intricately influenced by various factors, such as surface morphology, substrate orientation, and deposition process parameters. These factors play a crucial role in mitigating defects in the produced films, including decomposition, fluctuation, and layer interdiffusion.

During the initial stages of film growth, evaporated particles of the deposited material undergo absorption and chemical diffusion processes. The fate of these particles is determined by surface morphology; they may either be reflected or absorbed into the substrate surface. Absorption, in particular, involves the interaction between the evaporated particles and the substrate surface, characterized by a sticking coefficient representing the ratio of absorbed particles to the total amount deposited [1].

Absorption plays a pivotal role in shaping the crystallinity and microstructure of the resulting films. It encompasses two distinct forms: physical absorption and chemical absorption, which differ in the strength of atomic interaction. The interplay between these interactions can be elucidated through the Lennard–Jones curve, which describes the repulsive and attractive forces between atoms [1].

**Figure 1:**
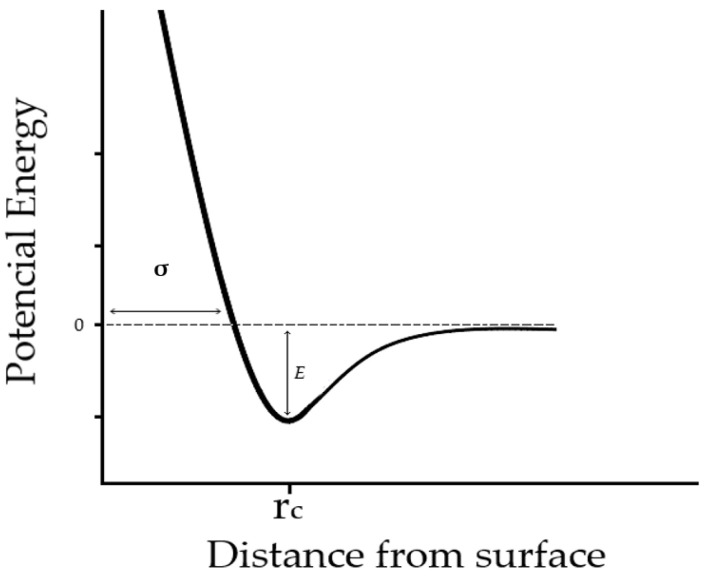
Lennard–Jones curve.

Absorption plays a pivotal role in shaping the crystallinity and microstructure of the resulting films. It encompasses two distinct forms: physical absorption and chemical absorption, which differ in the strength of atomic interaction. The interplay between these interactions can be elucidated through the Lennard–Jones curve (Figure 1), which describes the repulsive and attractive forces between atoms [1].

Typically characterized by two minima, the Lennard–Jones curve illustrates the attractive and repulsive forces between atoms, corresponding to the minimum potential energy of interaction. The first minimum signifies long-range attractive interactions, while the second represents repulsive forces arising from electron cloud overlap around the atoms [1].

(1) $$V(r)=4\varepsilon [{\left(\frac{\sigma }{r}\right)}^{12}-{\left(\frac{\sigma }{r}\right)}^{6}]$$

The combined effect of the minima in the Lennard–Jones curve results in a potential well with a minimum at a specific separation distance, which is crucial for determining molecular interaction stability. The depth of this potential well (ε in the Lennard–Jones curve) signifies the strength of attractive forces, while the position of the minima (σ in the Lennard–Jones curve) defines the range of interaction.

In physical absorption, particles are attracted to the surface through attractive interactions, characterized by an absorption potential (Ep). As evaporated particles of the deposited material approach the surface, they lose kinetic energy and form bonds with surface atoms, thereby reducing free surface energy. On the other hand, chemical absorption involves the creation of chemical bonds between the electrons of evaporated particles and substrate atoms, denoted by an absorption potential (Ec).

**Figure 2:**
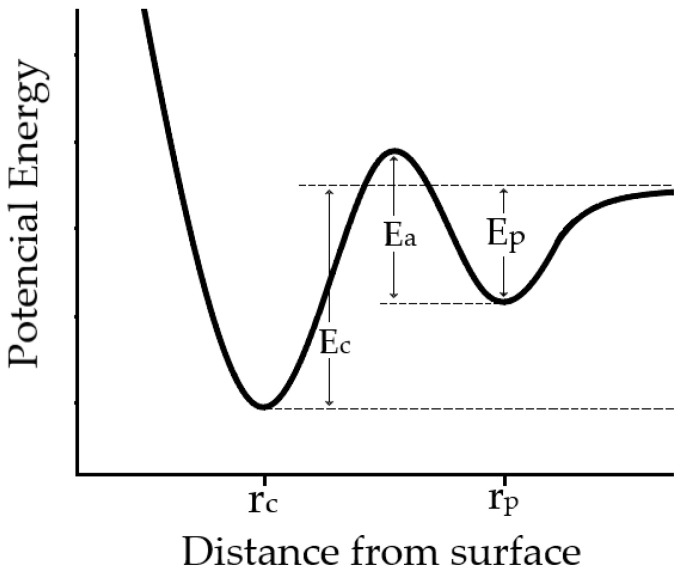
Physical absorption as an attractive interaction.

The process of physical and chemical absorption can be visualized as a function of distance versus potential. Optimal physical absorption occurs at a greater distance from the surface compared to chemical absorption. The disparity between these two stages is delineated by the effective energy barrier (Ea) (Figure 2).

**Figure 3:**
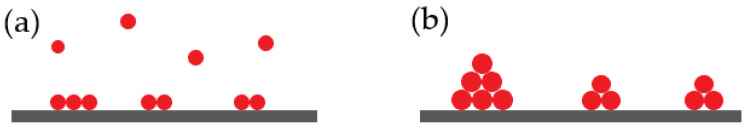
Volmer–Weber model: (**a**) creation of clusters; (**b**) clusters of deposited material.

In the Volmer–Weber growth mechanism (refer to Figure 3), material atoms are initially present as clusters in the vapor phase and subsequently condense onto the substrate, forming a thin film [2]. This growth process takes place at the atomic or molecular level and is significantly impacted by various substrate parameters, including temperature, deposition rate, and the properties of the deposited material, such as compatibility. The Volmer–Weber growth mechanism has proven to be advantageous in the fabrication of thin films for a range of applications, including semiconductor devices like transistors, solar cell units, and optical films [2].

**Figure 4:**
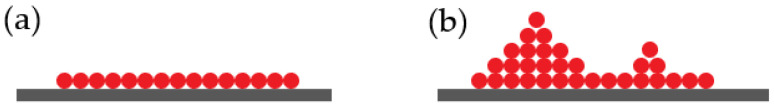
Frank–van der Merwe model: (**a**) creation of monolayer; (**b**) 3D islands of deposited material.

In the van der Merwe mechanism (see Figure 4), thin film material is deposited onto the substrate in the form of small particles generated through nucleation and growth processes in the vapor phase [3]. These particles subsequently adhere to the substrate, where they further develop into a continuous film. The growth of these particles is contingent upon factors such as the rate of material supply to the substrate, substrate temperature, and the properties of the metal material involved [3].

Absorbed particles subsequently aggregate into clusters of absorbed atoms, initiating the formation of three-dimensional (3D) islands. These islands then evolve into monolayers of deposited material through a process known as nucleation. Prior to nucleation, the growth of 3D islands is accompanied by the accumulation of stress, particularly tension, during the initial film or monolayer deposition [3].

In the context of layer deposition, the first layer holds paramount importance. Its characteristics are delineated by crucial particle size and an essential energy barrier, or Gibbs function. These parameters dictate the initial stage of film growth and profoundly influence subsequent layer formation and film properties (2).

(2) $$\Delta G_{\mathrm{total}}=\frac{4}{3}\pi r^{3}\Delta Gv+4\pi r^{2}\Upsilon$$

In the context of thin-film growth, Gibbs free energy plays a crucial role in determining the feasibility of the deposition process. Gibbs free energy (ΔG) is a fundamental concept in thermodynamics, representing the maximum amount of work that can be obtained from a system at constant temperature and pressure.

In the context of thin-film deposition, Gibbs free energy change determines whether the process is energetically favorable or not. If ΔG < 0, the process is spontaneous and can proceed without the input of external energy. If ΔG > 0, the process is non-spontaneous and requires the input of energy to occur.

For the growth of the first layer in thin-film deposition, Gibbs free energy plays a critical role in determining the feasibility of nucleation and adhesion of the deposited material to the substrate. The decrease in Gibbs free energy associated with the formation of the first layer indicates the stability of the system and the likelihood of successful film growth.

This compatibility criterion is elucidated by Young’s equation [4], a fundamental principle in the science of wetting that describes the relationship between surface tensions at the interface of a liquid–solid system. Young’s equation provides a simple yet elegant representation of the forces involved in wetting phenomena, facilitating an understanding of various practical applications such as adhesion and the behavior of different materials [4].

(3) $$\Upsilon_{fs}\;<\;\Upsilon_{fs}+\Upsilon_{vs}\times \cos ϴ$$

Young’s Equation (3) relates the pressure difference across the curved surface to the tension on the surface.

(4) $$\Upsilon_{fs}\;<\;\Upsilon_{fs}+\Upsilon_{vs}$$

(5) $$\Upsilon_{fs}=\Upsilon_{fs}+\Upsilon_{vs}$$

The Young equation stipulates that optimal monolayer growth occurs when the contact angle approaches 0 degrees or initiates within the range of 0 to 90 degrees [4]. This phenomenon, known as epitaxy, encompasses two intricate processes: homoepitaxy, where the deposited layer mirrors the material composition of the substrate, and heteroepitaxy, where the deposited layer features a distinct material composition from the substrate [4].

Homoepitaxy refers to a film that shares the same crystallographic orientation and composition as the substrate, effectively extending the substrate itself [5,6]. Conversely, in heteroepitaxy, the substrate closely matches the orientation of the deposited monolayer. When particles are absorbed onto the substrate, surface energy decreases as the active surface area diminishes [7]. However, the deposited monolayer typically possesses a distinct chemical structure from the substrate. Preferential binding of absorbent particles to the substrate rather than to each other occurs when lattice parameters are equal to or very similar to the deposited material. When lattice constants differ, it often results in the formation of stress within the deposited layer, which in turn promotes the growth of island-layer structures rather than a continuous monolayer [7].

(6) $$\Delta G*=\{\frac{16\pi \Upsilon vf}{3(\Delta Gv+\omega^{2})}\}\{\frac{2-3\cos ϴ+{cos(ϴ)}^{3}}{4}\}$$

The growth of a monolayer induces stress due to differences in lattice parameters, leading to the accumulation of strain potential. This strain potential amplifies as more monolayers stack up. As this strain potential increases beyond a critical value and the strain energy of the deposited layer surpasses surface tension, subsequent layers can form above the initially deposited one. During deposition, various defects may arise, consequently elevating the overall Gibbs energy. Consequently, the size of the initially deposited layer decreases, attributed to a reduction in the energy barrier of initial absorption (6) [7].

Nucleation and the simultaneous combination of monolayers culminate in the formation of the final microstructure of the thin film. The morphology of this structure is influenced by substrate temperature, substrate morphology, deposition rate, and surface diffusion. By manipulating or combining these factors, the resulting film morphology can be categorized into four subclasses: Z1, ZT, Z2, and Z3 [8].

As the film continues to grow, it begins to form basic structural zones, which are not uniformly distributed across its surface, with thickness varying due to blurred transitions between zones. Sometimes, distinguishing between these zones proves challenging. There are four fundamental structural zone models—Z1, ZT, Z3, and Z4 [8]—dependent on crucial parameters such as the ratio of normalized temperature (Ts) to surface diffusion (Tm) and the energy transfer of atoms at high energy. The transition between Z1 and Z4 models heavily relies on this ratio. For instance, the Z1 model predominates when the Ts/Tm ratio is low (<0.1 Pa) compared to surface diffusion. Surface diffusion manifests as columns approximately 10–20 nm in diameter forming on the substrate surface, separated by small gaps several nanometers wide. On this particular structure, an array of cones is superimposed, with the cones coalescing into domains across the surface, the size of which increases with thickness [2].

This mode closely resembles Z1 (with Ts/Tm~0.1 Pa), characterized by negligible surface diffusion. However, unlike Z1, this mode lacks the presence of gaps and domain cones. Z1 mode often results in significant porosity, leading to issues such as leakage, low electrical conductivity, and inhomogeneity, which are generally undesirable. However, porosity can be advantageous in certain technological applications, such as gas leakage detectors, catalytic reactions, and light absorption [2].

As the ratio of normalized temperature to surface diffusion (Ts/Tm) increases (Ts/Tm > 0.3 Pa), the system transitions into the Z2 mode. In this mode, surface diffusion becomes increasingly significant. Column formation in Z2 mode is characterized by tight grain boundaries between the columns. The diameter of these columns increases with the normalized temperature and surface diffusion ratio, resulting in a more refined crystalline structure. The transition from Z1 to Z2 modes is more pronounced at higher temperatures [2].

In the Z3 mode (Ts/Tm > 0.5), the ratio is even larger compared to the previous modes, indicating stronger surface diffusion. The surface of Z3 mode appears smooth, albeit with slight grooves across its surface. The morphology of this mode is characterized by equiaxed grain crystals, with the size of these crystals being proportional to the thickness of the deposited film [2].

## 3. Modeling Techniques

### 3.1. Quantum Mechanical Modeling

Quantum mechanical modeling is a powerful theoretical framework used to study the electronic structure and properties of magnetic films on substrates. It provides a detailed understanding of the behavior of electrons in the system, allowing for the calculation of energy levels, wave functions, and electronic interactions [9].

Quantum mechanical models, rooted in the foundational principles of physics, offer unparalleled accuracy in characterizing the behavior of individual electrons and their intricate interplay [10]. This precision is paramount for capturing nuances in magnetic interactions within films, encompassing attributes such as spin orientations, exchange interactions, and magnetic anisotropy [11]. Quantum mechanical frameworks provide a microscopic lens to fathom the underlying quantum states and electron distributions that underlie magnetic properties. This understanding extends to phenomena like magnetism switching, domain wall movement, and magnetic hysteresis, fostering deeper comprehension [11]. The predictive capability of quantum mechanical simulations guides the design and exploration of novel materials and configurations. By envisaging uncharted magnetic film properties, these models expedite material discovery and optimization, propelling advancements in magnetic-based technologies [12]. Quantum mechanical simulations adeptly encapsulate phenomena like tunneling and superposition, critical for decoding and manipulating magnetic behavior on the nanoscale. Such effects hold particular relevance in domains such as quantum computing and nanoscale magnetic devices [12].

**Figure 5:**
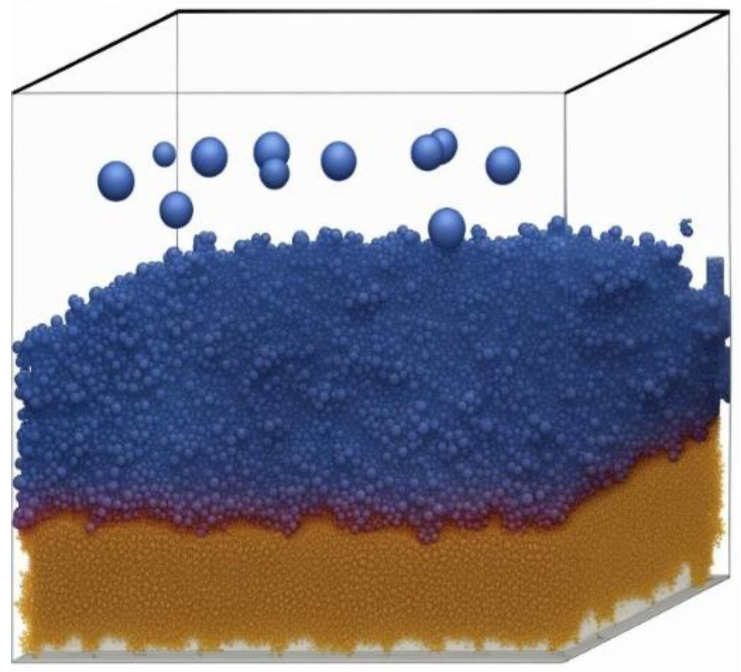
Simulated TiO_2_ thin-film growth.

The computational demands and time constraints associated with quantum mechanical calculations, particularly for extensive systems or prolonged time frames, present challenges. These limitations curtail the size of systems amenable to accurate modeling and the temporal extents that can be simulated. To accommodate the intricacies of real-world scenarios, quantum mechanical models often necessitate approximations and abstractions [12]. These expedients can introduce discrepancies, particularly in instances featuring robust correlations, intricate geometries, or extreme conditions. Magnetic films often encompass a medley of materials, spanning multiple elements, defects, and interfacial regions. Accommodating these multifaceted characteristics through quantum mechanical modeling demands advanced theoretical and computational approaches. The efficacy of quantum mechanical models hinges on parameters gleaned from empirical observations or other sources [12]. The sensitivity of model predictions to these parameters can engender uncertainties in the resultant outcomes. While quantum mechanical models yield precise numerical outcomes, their intuitive interpretation of the underlying physical mechanisms might not always be readily discernible. Extracting meaningful insights may necessitate a comprehensive grasp of quantum physics principles (Figure 5).

**Figure 6:**
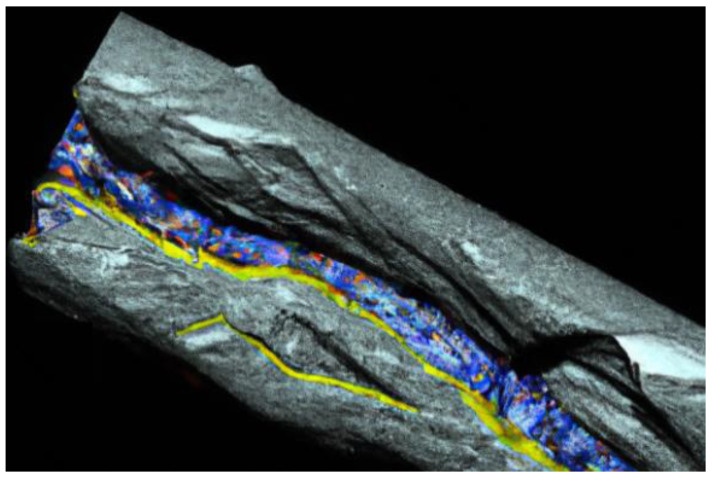
Simulated film substrate interface.

Central to the efficacy of quantum mechanical modeling is its prowess in explicating electronic structure and intermolecular dynamics within materials with unparalleled precision (Figure 6). The dimensionality-restricted nature of thin films confers upon them distinctive electronic traits that are divergent from their bulk counterparts. This elucidation augments our fundamental understanding of thin-film phenomena and concurrently stimulates the conceptualization of innovative devices endowed with bespoke functionalities. Moreover, quantum mechanical modeling furnishes a virtual crucible for unraveling the mechanistic, thermal, and optical attributes of thin films. The gamut of thermal transport phenomena, pivotal in nanoscale heat management, can be meticulously unveiled, thereby channeling the development of thermally conductive materials with elevated efficiency. Furthermore, the optical signatures inherent to thin films, including absorption, reflection, and emission, can be meticulously foretold, thereby engendering the design of advanced optical coatings and sensors with tailored light manipulation capabilities.

Quantum mechanical modeling also enables the calculation of various electronic properties of magnetic films on substrates [13]. These properties include electric bands, structure, density of states, magnetic moments, and magnetic entropy. Understanding surface changes can provide insight into the film’s magnetic behavior, its response to external magnetic fields, and the influence of the substrate on the electronic structure [13]. Confluence between experimental endeavors and quantum mechanical modeling can potentially orchestrate transformative shifts across myriad domains.

Quantum mechanics methods combined with micromagnetic simulations enable the exploration and realization of novel spintronic device concepts, such as skyrmion-based devices, spin-wave devices, and topological insulator-based devices, with unique functionalities and enhanced performance [14].

Additionally, quantum mechanical modeling can provide information about film–substrate interfaces. It allows for the instigation of bonding characteristics, charge transfer, and interfacial electronic states. Quantum mechanical modeling, particularly DFT calculations [15], has been widely used in the study of magnetic films on substrates. By accurately capturing the electric structure and its properties, this approach provides valuable insight and predictions, guiding experimental investigations and the design of magnetic materials and devices [15]. Quantum mechanical modeling is a vital tool for understanding the behavior of magnetic films on substrates at the atomic and electronic levels. It offers a theoretical foundation for studying electronic structures, magnetic properties, and interfacial effects, contributing to the advancement of magnetic film technology [16] (Table 1).

Electronic structure calculations are computational methods used in quantum mechanical modeling to predict and analyze the behavior of atoms, molecules, and materials at the atomic and molecular level. These calculations aim to solve the Schrödinger equation, which describes the behavior of quantum mechanical systems, particularly the motion of electrons within atoms and molecules. The Schrödinger equation, however, cannot be solved analytically for systems with more than one electron, except for a few simple cases. Therefore, electronic structure calculations rely on approximations and numerical methods to find solutions that are accurate enough to be useful for understanding chemical and physical properties.

The motivation behind electronic structure calculations lies in their ability to provide detailed insights into the electronic properties of matter. Some of the advantages of using electronic structure calculations include:

Electronic structure calculations allow scientists to gain a profound understanding of chemical bonding in molecules and materials. By analyzing the distribution of electrons and their interactions, researchers can predict molecular geometries, bond strengths, and reactivity. These calculations can accurately predict various molecular properties such as energy levels, electronic spectra, dipole moments, and polarizabilities. In materials science, electronic structure calculations play a pivotal role in predicting and designing materials with desired properties. By calculating electronic band structures, density of states, and other electronic properties, researchers can identify materials with specific electronic and optical characteristics for various applications, including electronics, photovoltaics, and catalysis. Electronic structure calculations provide insights into the mechanisms of chemical reactions by simulating the electronic structure of reactants, intermediates, and transition states. Electronic structure calculations can complement experimental observations by providing detailed insights into the underlying electronic structure of molecules and materials. Through computational methods, researchers can explore a wide range of molecular and material properties without the need for expensive and time-consuming experimental procedures. Electronic structure calculations can be applied to a diverse range of systems, from simple molecules to complex materials.

In the realm of quantum mechanics, a distinct avenue of inquiry involves the formulation of theoretical frameworks aimed at scrutinizing the characteristics of intricate molecular systems, denominated as quantum molecular dynamics [17]. This approach relies upon the application of the Born–Oppenheimer approximation, postulating the segregation of variables into substantial nuclei and agile electrons. The quantum molecular dynamics methodology, as introduced by Car–Parrinello, entails the utilization of function minimization techniques [17].

The concept underlying Car–Parrinello molecular dynamics (CPMD) involves the simultaneous computation of electronic and atomic subsystems. The trajectory of nuclei is dictated by the ensemble of their respective coordinates {R_i_|i = 1,…,N_n_}. The electronic degrees of freedom are defined by a collection of quantum-mechanical wave functions {Ψj|j = 1,…,N_e_} [17].

(7) $$M_{i}{\ddot{R}}_{i}=-\frac{\partial E}{\partial R_{i}}=F_{i}$$

(8) $$\mu \ddot{\psi_{j}}(r,t)=-\hat{H}\psi_{j}(r,t)+\sum_{k}\wedge_{jk}\psi_{k}(r,t)$$

In the given context, the symbol Fi represents the resultant force exerted on the atom.
E and Ĥ represent the energy functional and the Kohn–Sham Hamiltonian.
μ denotes the fictitious electron mass.
$\wedge_{jk}$ stands for indefinite Lagrange multipliers.

The selection of the fictitious electron mass is aimed at expediting the convergence of the algorithm. In parallel, the Lagrange multipliers play a pivotal role in generating additional forces that enforce the orthonormality of the wave functions [17] (7,8).

In the Car–Parrinello theory, the total energy of a nanosystem is a function of the coefficients in the expansion of the electron wave function over a given basis. When specific coefficients are minimized, the system undergoes cooling and stabilization. The initial segment of expressions comprises classical Newtonian motion equations for a set of particles, which are iteratively solved until equilibrium is attained [18]. Consistency between the ion and electron subsystems is achieved through the concurrent minimization of the energy functional. It is important to note that this method does not portray the genuine dynamics of the nanosystem but rather simulates its fictitious evolution, leading to an equilibrium state with stable energy for a multi-electron particle system [18].

The Car–Parrinello molecular dynamics method is recognized for its ability to accurately replicate the properties of semiconductor and dielectric materials [19]. However, for metallic systems in close proximity to the band gap, where numerous states possess closely spaced eigenvalues, even a minor alteration in total energy can lead to significant fluctuations in electron density. Moreover, the applicability of the Car–Parrinello approach is constrained to a limited number of atoms in a nanosystem due to the intricate nature of solving the equations and the associated computational expenses [19].

An alternative to Car–Parrinello molecular dynamics is Born–Oppenheimer molecular dynamics (BOMD), founded on the Born–Oppenheimer approximation [20]. This method involves the segregation of the nuclear and electronic subsystem descriptions. The motion of the nuclei is governed by classical mechanical equations, while the energy and forces acting on them are computed by solving the Schrödinger equation for electronic wave functions at each time step. This approach is advantageous in managing the complexity of metallic systems and circumventing computational challenges associated with larger nanosystems [20].

(9) $$M_{i}{\ddot{R}}_{i}=-\nabla_{i}[\underset{\psi_{1},\ldots ,\psi_{N_{e}}}{\min}E(R_{1},\ldots ,R_{N_{n}};\;\psi_{1},\ldots ,\psi_{N_{e}})]$$

In the given context, the total energy (E) is subject to minimization through the optimization of electron wave functions at fixed nuclear coordinates [21]. Typically, this minimization is achieved by solving the Kohn–Scham equations or employing multidimensional optimization algorithms (9). The BOMD method, which involves the segregation of nuclear and electronic subsystem descriptions, proves highly accurate and can effectively describe the properties of dielectrics and metals. However, both BOMD and CPMD methods incur substantial computational costs, limiting their application to smaller nanosystems [21].

To address this limitation and enhance the capacity of the system under investigation, efforts have been directed toward the development of methods that combine classical molecular modeling with electronic structure calculations [21]. This approach is exemplified by embedded atom potentials such as the Embedded Atom Model (EAM) and Modified Embedded Atom Method (MEAM). The Embedded Atom Method is derived from theoretical considerations of the electron density functional and describes nanosystem behavior through a set of equations, offering a means to reduce computational complexity and extend its applicability to larger systems [21]. The EAM originates from the theoretical principles of the electron density functional and articulates the behavior of a nanosystem through the following set of equations:

(10) $$M_{i}{\ddot{R}}_{i}=-\frac{\partial E}{\partial R_{i}}=F_{i},$$

(11) $$U_{i}=F\left(\rho_{\dot{i}}\right)+\sum_{J;1\cdot \neq j}\phi {\left(\left|R_{i}-R_{j}\right|\right)}_{.}\;\;$$

(12) $$\rho_{\dot{i}}=\sum_{J;1\cdot \neq j}\Psi {\left(\left|R_{i}-R_{j}\right|\right)}_{.}\;$$

where F(ρ_i_) is an immersion function that characterizes the interaction of an individual atom with a medium possessing electron density ρ_i_. ϕ(|R_i_–R_j_|) is pair potential. The electron density created by the j-th atom at the location of the i-th atom (10–12).

The BOMD method is highly accurate and effective in describing the properties of materials such as dielectrics and metals [21]. However, both BOMD and CPMD methods come with significant computational costs, making them primarily suitable for small-sized nanosystems. The high accuracy of quantum mechanics methods is intricately tied to the complexity of the algorithms and approaches employed, representing a limitation of this theoretical framework [21].

To address the challenges posed by the complexity of quantum mechanics models, various assumptions and modifications have been introduced to eliminate limitations and reduce the complexity and dimensionality of solved problems by limiting the degrees of freedom of the investigated objects. Key approximations in first-principles methods include the Born–Oppenheimer approximation, which assumes the separation of variables into massive nuclei and fast electrons, and the Hartree–Fock approximation, which transforms the dependence of the Schrödinger equations on the multi-electron wave function into a correlation relation of one-electron functions.

A significant aspect of quantum mechanics methods is occupied by the theory of electron density functional, which addresses the issue of increasing complexity and dimensionality as the number of observed particles rises. In this approach, the energy state of a nanosystem involving nuclei and electrons interacting in an external field is described as a functional that depends on the particle density. Evolutionary changes and various descriptions of exchange-correlation energy, including Thomas–Fermi theory, Hohenberg–Kohn theorems, Kohn–Sham formalism, local density approximation, generalized and meta-generalized approximations, and hybridization of the generalized gradient method, underscore the relevance and importance of electronic density functional theory and showcase the ongoing development of this method.

In one study conducted by Hegedus and Kugler (2005), quantum mechanical simulation was utilized in order to stimulate the dynamic interatomic interaction of selenium molecules [22]. This model is then used to simulate the growth of amorphous selenium thin films. Though the model has limitations, it seems most realistic to simulate the behavior of amorphous selenium [22].

**Figure 7:**
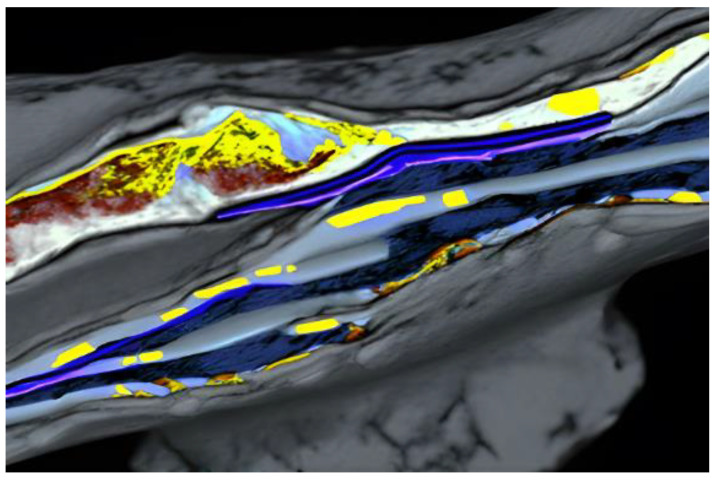
Simulated magnetic thin film.

Another study is related to the simulation of Al_2_O_3_ thin films by utilizing quantum mechanical simulation (Figure 7). The study was conducted by Turowski et al. (2015) [23]. In this study, several simulation techniques, including quantum modeling simulation, were applied in order to simulate the scattering process and, therefore, optimize it. The developed virtual coater concepts demonstrated remarkable performance in this investigation. Furthermore, the method holds considerable promise for its application and study of diverse layer materials and coating processes of interest beyond Al_2_O_3_ thin films [23].

The next study conducted by the same author focused on the simulation of thin-film technology for TiO_2_ [24]. The paper introduced an extension to the virtual quoted concept, wherein the determined optical properties are linked with rare acquisition models. This contribution highlights the significant potential of atomistic simulation techniques in advancing our understanding and predictive capabilities in thin film and coaching process-related domains, operating avenues of human research, and technological adventures in the field [24].

The exploration of magnetic materials and their behavior has grown increasingly significant across a spectrum of technological domains, from data storage and medical imaging to energy conversion and sensing applications [25]. Within this context, the utilization of quantum mechanical modeling has emerged as a potent avenue to unravel the intricate dynamics of magnetic films. This approach delves into the fundamental quantum properties of electrons, their interactions, and the resulting magnetic phenomena. As with any modeling methodology, employing quantum mechanics to study magnetic films comes with a set of inherent advantages and corresponding disadvantages [25].

### 3.2. Monte Carlo Simulations

While quantum mechanical modeling provides essential insight into the electronic structure, classical modeling approaches are often employed to study the dynamics and thermal fluctuation of magnetic films on substrates. These approaches allow for the simulation of large-scale systems and longer time scales. This technique is particularly well-suited for studying systems characterized by randomness, uncertainty, and nonlinearity, making it indispensable for deciphering phenomena ranging from quantum mechanics and statistical physics to complex biological processes.

Monte Carlo simulation utilizes random sampling methods to study the thermodynamic properties and thermal fluctuations of magnetic films on substrates [26]. By considering temperature, exchange interaction, and anisotropy effects, Monte Carlo simulations can explore the phase transition, domain formation, and magnetization dynamics in the system. Monte Carlo simulations also excel at elucidating the behavior of large and complex systems that defy traditional analytical approaches. These simulations provide statistical information that enables the investigation of the temperature-dependent properties of film–substrate systems [26].

**Figure 8:**
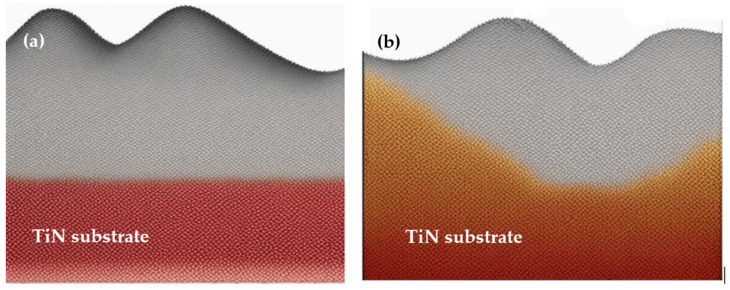
Simulated formation of thin films (**a**) on TiN substrate (**b**) different film formation.

Monte Carlo simulation calculates the depositing process by tracking the movement and interaction of individual atoms or molecules [27]. The simulations consider parameters such as incident particle flux, energy, and surface interactions to determine the probability of deposition at various sites on the substrate. By performing numerous interactions on these stochastic processes, Monte Carlo stimulations provide statistical information about the resulting film’s growth, including its microstructure, morphology, and surface roughness [27]. Through Monte Carlo simulations, researchers can investigate the effect of deposition conditions such as temperature, pressure, and induced flux on thin-film growth. These simulations allow for the exploration of various deposition parameters and their impact on film properties. Additionally, Monte Carlo simulations can provide insight into the nucleation, island growth, coalescence, and other processes that occur during the thin-film deposition (Figure 8) [28].

The Monte Carlo Metropolis algorithm offers a natural approach for simulating temperature effects in scenarios where dynamic considerations are unnecessary, owing to its rapid convergence to equilibrium and its relatively straightforward implementation [29].

In the context of a classical spin system, the Monte Carlo Metropolis algorithm unfolds in the following manner: Initially, a stochastic selection is made of a specific spin index i, followed by a random alteration of its initial spin direction S_i_ to a novel trial configuration S_0i_, denoted as a “trial move” [29]. Subsequently, the alteration in energy ΔE = E(S^’^_i_) – E(S_0i_) between the pre-existing and updated states is assessed, and the trial move is accepted with a probability determined by a specified criterion [29].

(13) $$P=\exp\left(-\frac{\Delta E}{K_{b}T}\right)$$

The acceptance decision is made by comparing the calculated probability with a random number drawn from a uniform distribution within the interval [0, 1] (Equation (13)) [29]. Probabilities exceeding 1, indicative of an energy reduction, are unconditionally approved. This iterative process is repeated until a total of *N* trial moves have been undertaken, where *N* represents the count of spins within the entire system. Each collection of *N* trial moves constitutes a single iteration of the Monte Carlo algorithm [29].

The character of the trial move holds significance in light of two essential requirements for any Monte Carlo algorithm: ergodicity and reversibility. Ergodicity dictates that all possible states of the system should be accessible, while reversibility necessitates that the transition probability between two states remains invariant, explicitly expressed as Equation (14) manifests the evident reversibility as P(S_i_ → S^’^_i_) = P(S_i_ → S^’^_i_), where the probability of a spin change is contingent solely upon the initial and final energy [30].

Ergodicity can be readily satisfied by relocating the chosen spin to a random position on the unit sphere. However, this approach yields an undesirable outcome at low temperatures, where substantial deviations of spins from the collinear direction become highly improbable due to the strength of the exchange interaction [31]. Consequently, at low temperatures, a sequence of trial moves on the unit sphere often results in a majority of moves being rejected. Ideally, a higher acceptance rate of approximately 50% is sought, as excessively high or low rates necessitate a significantly greater number of Monte Carlo steps to achieve a state representative of genuine thermal equilibrium [31].

A highly effective Monte Carlo algorithm for classical spin models was introduced by Hinzke and Nowak [32]. This algorithm employs a combinational approach, incorporating a mix of various trial moves. A key strength of this method lies in its ability to efficiently sample the entire available phase space while sustaining a reasonable acceptance rate for trial moves. The Hinzke–Nowak method specifically employs three distinct types of moves, i.e., spin flip, Gaussian, and random [32].

The spin flip move involves a straightforward reversal of the spin direction, denoted as S^’^_i_ = −S_i_, explicitly facilitating the initiation of a switching event. This move is analogous to a step in Ising spin models [33]. It is important to note that spin flip moves, in isolation, do not inherently satisfy ergodicity in classical spin models, as states perpendicular to the initial spin direction are inaccessible. However, when combined with other ergodic trial moves, this limitation is effectively addressed [33].

The Gaussian trial move takes the initial spin direction and relocates the spin to a point on the unit sphere near the initial position. This relocation is determined by the following expression:

(14) $${S’}_{i}=\frac{{S’}_{i}+\sigma_{g}\Gamma }{\left|{S’}_{i}+\sigma_{g}\Gamma \right|}$$

In the expression, Γ  represents a Gaussian-distributed random number and $\sigma_{g}$ is the width of a cone around the initial spin $S_{i}$ [34]. Following the generation of the trial position, the position is normalized to ensure a spin of unit length. The selection of a Gaussian distribution is intentional, as, after normalization, the trial moves exhibit uniform sampling over the cone [34]. The width of the cone is typically chosen to be temperature-dependent and adheres to the following form:

(15) $$\sigma_{g}=\frac{2}{25}{\left(\frac{K_{b}T}{\mu_{b}}\right)}^{1/5}$$

Therefore, the Gaussian trial move tends to favor small angular changes in the spin direction at low temperatures, resulting in a favorable acceptance probability across a broad range of temperatures. The concluding random trial move selects a random point on the unit sphere according to the following formula:

(16) $${S’}_{i}=\frac{\Gamma }{\left|\Gamma \right|}$$

This selection ensures ergodicity for the entire algorithm, guaranteeing efficient sampling of the phase space, particularly at elevated temperatures. In each trial step, one of these three trial moves is randomly chosen, generally resulting in favorable algorithmic properties [34].

One of the fundamental components of Monte Carlo simulation is the Metropolis algorithm, introduced by Nicholas Metropolis et al. in 1953 [35]. Metropolis algorithms are versatile and can be adapted to simulate systems with complex energy landscapes, making them suitable for a wide range of applications. These algorithms are particularly efficient for simulating systems with a large number of degrees of freedom, where traditional methods may struggle due to computational complexity. Metropolis algorithms are relatively straightforward to implement compared to some other Monte Carlo techniques, which enhances their accessibility to researchers and practitioners. Under certain conditions, Metropolis algorithms exhibit desirable convergence properties, ensuring that the simulation results approach the true distribution of the system being modeled. Metropolis algorithms can be applied to diverse problems, including equilibrium statistical mechanics, optimization, and Bayesian inference, providing a unified framework for tackling different types of simulation tasks.

In some cases, Metropolis algorithms may exhibit slow convergence or high autocorrelation between samples, especially in systems with strong correlations or long-range interactions. Metropolis algorithms often require careful tuning of parameters such as the proposal distribution width or acceptance criteria, which can be nontrivial and may impact simulation efficiency. For systems with a finite number of particles or degrees of freedom, finite-size effects can influence the simulation results, leading to deviations from the true behavior of the system, particularly near-phase transitions. In high-dimensional parameter spaces, Metropolis algorithms may encounter difficulties in efficiently exploring the state space, potentially resulting in poor sampling efficiency or biased estimates. While suitable for many problems, Metropolis algorithms may not scale well to extremely large systems or high-dimensional parameter spaces due to computational constraints.

Quantum Monte Carlo (QMC) methods are computational techniques used in quantum chemistry and condensed matter physics to solve the electronic structure of atoms, molecules, and materials [36]. These methods excel in systems where strong electronic interactions cannot be accurately described using perturbative methods like Hartree–Fock theory or density functional theory. Quantum Monte Carlo methods utilize Monte Carlo integration techniques to solve the Schrödinger equation for many-body systems. Instead of directly solving the wavefunction, they typically work with the probability distribution of particles in configurational space, providing an accurate description of the electronic structure by sampling this space stochastically [36]. Variational Monte Carlo (VMC) uses a trial wavefunction to approximate the ground state wavefunction, approaching the exact ground state energy by optimizing the trial wavefunction’s parameters. Diffusion Monte Carlo (DMC) evolves a population of random walkers in imaginary time to project out the ground state from a trial wavefunction. Particularly effective for treating large systems with accurate electron–electron correlation [36]. Quantum Monte Carlo with Slater Determinants (QMC–SD) utilizes Slater determinants to represent the trial wavefunction and employs Monte Carlo techniques to calculate the expectation values of observables [37].

Green’s Function Monte Carlo (GFMC) uses the imaginary-time Green’s function to propagate a set of walkers, providing highly accurate results but demanding computational resources. Quantum Monte Carlo methods offer several advantages, including high accuracy, especially in treating strong electron correlation effects, and well-defined convergence properties [37]. However, they also face challenges such as computational cost, statistical errors, and the fermion sign problem, particularly in systems with fermions. These methods find applications in computational chemistry, studying molecular properties, reaction mechanisms, and material properties [38]. They are also used in condensed matter physics to investigate the electronic structure of solids, including strongly correlated electron systems, superconductivity, and magnetism. Overall, Quantum Monte Carlo methods represent a powerful approach for accurately describing the electronic structure of complex systems, continually advancing with improvements in algorithms and computing power, making them invaluable tools in theoretical and computational chemistry and physics [38].

An example of using Monte Carlo with a combination of molecular dynamic simulations was a study conducted by Namakian et al. (2021) [39]. In this study, sequential molecular dynamic time-stepped force bias Monte Carlo simulations were employed to simulate the deposition process and illustrate the growth mechanism of Cu thin film on TiN substrates. The result of this study was a comparison of kinetic Monte Carlo simulations of 2D islands of Cu thin film on TiN substrates and a demonstration of behavior and characteristics observed during the initial stage of growth [39].

The next study by Prudnikov (2016) is related to the simulation of magnetic multilayer structures with the use of Monte Carlo simulations [40]. This study presented a description of giant magnetoresistance effects in magnetic multilayer structures. A tropical Heisenberg model is employed to determine the magnetic properties of the ferromagnetic films that constitute these structures [40]. Monte Carlo simulations are conducted to investigate the magnetic properties of structures composed of two ferromagnetic films separated by a nonmagnetic film. A novel methodology was developed in this study for determining magnetoresistance using the Monte Carlo method. This approach was employed to calculate the temperature dependence of magnetoresistance for both free layer and spin valve structures and the variant thicknesses of the ferromagnetic films [40].

A similar study with regards to the growth of ferroelectrics with the use of Monte Carlo simulation was conducted by Candia (2001) [41]. In this study, Monte Carlo simulations were employed as a primary computational tool to investigate the growth of magnetic films exhibiting ferromagnetic interaction between the nearest spins [41].

In the realm of magnetic film studies, Monte Carlo simulations have emerged as a preeminent computational tool, offering a sophisticated means to unravel the intricate dynamics governing these systems [42]. Employing a stochastic methodology, these simulations meticulously replicate the complex evolution of magnetic systems, thereby furnishing invaluable insights into their nuanced properties, intricate interactions, and expansive applicability [42].

Monte Carlo simulations are distinguished by their adeptness in capturing the statistical intricacies and averaging effects inherent in intricate magnetic systems [43]. Leveraging a myriad of random processes, these simulations yield precise estimates of macroscopic properties such as magnetization, susceptibility, and heat capacity. Furthermore, their capacity to probe relatively large and realistic systems, which may be beyond the purview of conventional methods like quantum mechanical modeling, renders them indispensable in the investigation of magnetic films harboring a multitude of atoms or exhibiting intricate structures [43]. The intrinsic incorporation of temperature effects within Monte Carlo simulations enables an in-depth exploration of temperature-dependent phenomena, including magnetic phase transitions and thermal fluctuations [44]. This capability is pivotal for comprehensively understanding the stability and behavioral nuances of magnetic films under diverse thermal conditions, thus enriching our insights into their practical applicability [44].

**Figure 9:**
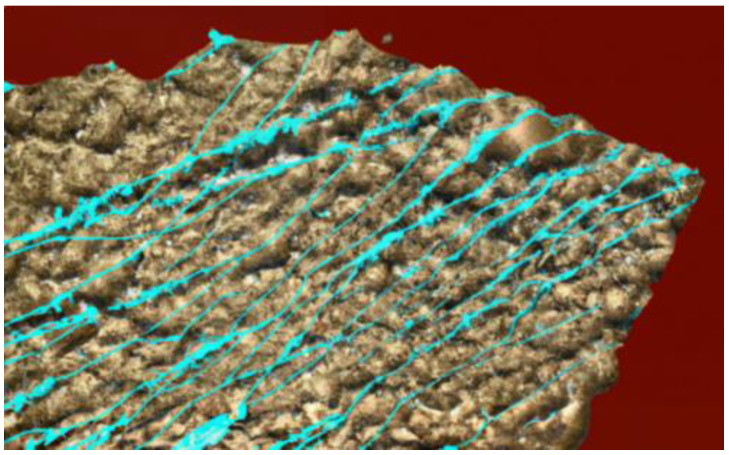
Simulated surface of a chromium oxide film.

Moreover, the accessibility and versatility of Monte Carlo simulations augment their utility, rendering them accessible to researchers across varying levels of expertise [44]. Widely embraced within the scientific community, Monte Carlo simulations are readily implementable through available software packages, further democratizing their usage and fostering widespread exploration in the field [44]. Facilitating a systematic exploration of parameter space, Monte Carlo simulations empower researchers to methodically investigate the influence of various parameters, such as exchange interactions, anisotropy constants, and external fields, on the magnetic behavior of films [45]. This comprehensive approach not only enhances our fundamental understanding but also paves the way for tailored designs and optimized functionalities in magnetic film applications. However, it is imperative to acknowledge that, akin to any modeling technique, the utilization of Monte Carlo simulations entails certain trade-offs and considerations. Assessing these merits and demerits judiciously in alignment with specific research objectives and computational capabilities is essential for maximizing their efficacy in elucidating the complex behavior of magnetic films (Figure 9).

By integrating Monte Carlo simulations with micromagnetic simulations, researchers can predict the performance of spintronic devices under realistic operating conditions, including temperature variations and external magnetic fields. This aids in device optimization and reliability assessment.

Monte Carlo simulations combined with micromagnetic simulations can predict the behavior of magnetic sensors with high accuracy. Quantum mechanics methods provide insights into the underlying physics governing spin-dependent phenomena, essential for designing sensitive and selective spin-based sensors for applications in magnetic field sensing, bio-sensing, and magnetic resonance imaging (MRI).

In the realm of computational modeling, Monte Carlo simulations stand as a formidable tool for unraveling the intricate dynamics of magnetic systems, although they are not without their challenges [45]. Monte Carlo simulations can be computationally intensive, particularly for systems with intricate interactions or when probing extended time scales. This computational demand can limit the feasibility of studying extremely large systems or conducting simulations over long periods. While providing statistical accuracy, Monte Carlo simulations often involve simplifications of the underlying physical interactions. These approximations can potentially affect the fidelity of results, particularly in cases where quantum mechanical effects play a crucial role. Monte Carlo simulations typically assume that the system reaches thermal equilibrium during the simulation [45].

However, this assumption may not hold in scenarios involving non-equilibrium processes or rapid changes in system dynamics. Monte Carlo simulations are generally classical in nature and may not fully capture quantum mechanical effects significant in certain magnetic systems. Quantum phenomena such as spin tunneling or entanglement may not be adequately represented in classical Monte Carlo simulations [46].

Interpreting the results of Monte Carlo simulations can be challenging, especially when dealing with complex systems. Extracting meaningful physical insights from the statistical data generated by simulations necessitates a profound understanding of both the simulation technique and the magnetic phenomena under investigation [46].

Despite these challenges, Monte Carlo simulations remain a powerful tool in the study of magnetic films, providing valuable insights into their behavior and properties. It is imperative for researchers to navigate these complexities with care, leveraging the strengths of Monte Carlo simulations while mitigating their limitations, to maximize the utility of this computational approach in advancing our understanding of magnetic systems [47].

By combining Monte Carlo simulations with experimental data and other modeling techniques, researchers can refine their understanding of thin-film growth mechanisms [48]. Optimization of the position processes and prediction of the properties of the resulting films improves the integration of simulation and experimentation and enables more efficient and targeted development of films for a wide range of applications [48] (Table 2).

Monte Carlo simulation and modeling of dynamic simulation shed light on dynamic behavior, thermal effects, and thermal phenomena. These modeling approaches play a crucial role in elucidating the magnetic properties, transport behavior, and response of magnetic films on substrates, thereby facilitating the design and optimization of magnetic devices.

### 3.3. Density Functional Theory Calculations

Density Functional Theory (DFT) calculations, a cornerstone of modern computational chemistry and physics, have emerged as a transformative approach with far-reaching implications for diverse scientific domains. Density functional theory (DFT) calculations are widely used computational methods in scientific research for investigating the properties of materials, including thin films. DFT provides very valuable insight into the electronic structure, chemical bonds, and physical characteristics of these films [49].

DFT is based on the principle that the electronic density contains all the necessary information about the system. By solving the Schrödinger equations for self-consistency, DFT allows for the determination of the ground-state properties and equilibrium configuration of the magnetic films [50].

The core principles of density-functional theory encompass the Hohenberg–Kohn and Kohn–Sham theorems. The original Hohenberg–Kohn and Kohn–Sham theorems readily lend themselves to expansive adaptation beyond their initial formulations, spanning a broad spectrum of physical scenarios.

In DFT calculations, the electric structure of the film substrate system is described by the electron density, which is governed by the Kohn–Sham equation [50] (Equation (17)) as follows:

(17) $${\hat{H}}_{ks}\Psi_{i}\left(r\right)=\left[-\frac{\hbar^{2}}{2m}\nabla^{2}+V_{eff}\left(r\right)\right]\Psi_{i}(r)=\varepsilon_{i}\;\Psi_{i}(r)$$

where:

$\Psi_{i}(r)$  is the wave function of the i electron.
$\varepsilon_{i}$  is the energy of the i electron.
$\nabla^{2}$  is the Laplacian operator.
$V_{eff}\left(r\right)$  is the effective potential, which includes the external potential due to the atomic nuclei and any additional external potential present in the system.

The Kohn–Sham equation is the fundamental equation in density function theory for the behavior of electrons in a material, including thin films [50]. This equation represents an effective single-particle problem, where the electrons move in an effective manner that includes the interaction with the atomic nuclei and the exchange-correlation potential. The exchange-correlation potential accounts for the effects of electron–electron interactions, which are challenging to describe exactly and are often approximated in practical calculations. The Kohn–Sham equation allows us to map the interacting many electron problems to a set of non-interacting single electron equations [50].

To solve the Kohn–Sham equations, various numerical technologies can be employed, such as blending of weight basis sets, localization of atomic orbitals, or real-space grids [50]. These techniques discretize the electronic wave functions and perform interactive calculations to converge onto the self-consistent solution [50].

The core principles of density-functional theory encompass the Hohenberg–Kohn and Kohn–Sham theorems. The original Hohenberg–Kohn and Kohn–Sham theorems readily lend themselves to expansive adaptation beyond their initial formulations, spanning a broad spectrum of physical scenarios.

The Landau–Lifshitz–Gilbert calculations for thin films involve several case steps. First, geometry optimization is performed to determine the optimized atomic position in the lattice parameters of the thin-film structure. This involves minimizing the total energy of the system by iteratively adjusting the atomic position until coherence is achieved [51]. The total energy is typically calculated using appropriate exchange-correlation functions, such as the generalization gradient approximation (GGA) or hybrid functionals. Next, the electronic structure calculation is conducted. Once the optimization geometry is obtained, the electronic structure of the thin film is computed [51]. This step involves solving the Kohn–Sham equations (Hohenberg–Kohn theorem), which describe the behavior of the electrons in the system. By solving this equation self-consistently (Equation (18)), information about the energy levels, band structure, and density of the states of the film can be obtained [52].

(18) $${\hat{H}}_{e}=\Sigma_{i}(-\frac{\hbar^{2}}{2m}\frac{\partial^{2}}{\partial {r_{i}}^{2}}-\Sigma_{I}\frac{Z_{I}e^{2}}{\left|r_{i}-R_{j}\right|})+\sum_{i<j}\frac{e^{2}}{\left|r_{i}-r_{j}\right|}$$

Here, ${\hat{H}}_{e}$ represents the electronic Hamiltonian. The first term represents the kinetic energy of electrons, with $\frac{\hbar^{2}}{2m}\frac{\partial^{2}}{\partial {r_{i}}^{2}}$ being the kinetic energy operator. The second term is the potential energy due to the interaction of the i-th electron with a nucleus located at Rj, given by $\frac{Z_{I}e^{2}}{\left|r_{i}-R_{j}\right|}$. The third term is the potential energy due to the electron–electron interaction, given by $\frac{e^{2}}{\left|r_{i}-r_{j}\right|}$ for all distinct pairs i,j of electrons.

In DFT, the electron density ρ(r) serves as the fundamental variable, in contrast to the many-body wave function used in traditional quantum mechanics [53]. The electron density is considerably more manageable than the many-body wave function since it depends on only three spatial coordinate variables, irrespective of the number of electrons in the system. This simplification has been justified by the Hohenberg–Kohn theorem, which establishes a one-to-one correspondence between the external potential and the ground-state electron density, rendering the electron density a sufficient and convenient descriptor for the quantum system [53].

The Hohenberg–Kohn theorem establishes a one-to-one correspondence between the ground-state electron density and the external potential [54]. Consequently, if the ground-state electron density is known, the external potential is uniquely determined. Furthermore, the physical properties associated with the ground-state wave function can, in principle, be unambiguously derived from the electron density [54]. Specifically, the kinetic and electron–electron interaction energies of the ground state can be expressed as universal functionals of the electron density, denoted as E_kin_[ρ] and E_ee_[ρ], respectively. The term “universal” indicates that the functional forms are independent of the specific external potential [54].

The theorem also provides a variational principle. When we define the following energy functional:

(19) $$E_{v}[\rho ]=E_{kin}[\rho ]+\int \rho \left(r\right)v\left(r\right)dr+E_{ee}[\rho ]$$

for some external potential *V*(r), the functional satisfies the inequality as follows:

(20) $$E_{v}\left[\rho \right]\geq E_{v}\left[\rho_{0}\right]=E_{0}$$

where *ρ*_0_ (*E*_0_) is the ground-state electron density (energy) under the potential *V*(r), respectively. Therefore, the ground-state electron density can be obtained by searching for the electron density that minimizes the energy functional $E_{v}\left[\rho \right]$ [55].

Although the variational principle in Equation (19) appears simple, a significant challenge lies in the fact that the exact forms of the functionals, E_kin_[*ρ*] and E_ee_[*ρ*], are unknown. To address this issue, Kohn and Sham introduced the concept of “orbitals” to approximate the kinetic energy functional E_kin_[*ρ*]. This innovative approach has paved the way for performing DFT calculations with sufficient accuracy for practical applications [55].

Therefore, the majority of modern DFT implementations utilize the Kohn–Sham scheme. We will delve into this scheme in more detail below. Regarding the direct variational approach, known as orbital-free DFT, which is less accurate compared to the Kohn–Sham approach but offers the advantage of faster computations, current research efforts are focused on constructing accurate kinetic energy functionals. One strategy involves striving to reproduce the Kohn–Sham kinetic energy as accurately as possible [55].

The Kohn–Sham scheme introduces an auxiliary non-interacting system designed to yield the same electron density as that of the interacting system. The non-interacting system is described by the single-particle Schrödinger equation, commonly referred to as the Kohn–Sham equation, as follows:

(21) $$\left\lceil -\frac{\hbar^{2}}{2m}\frac{\partial^{2}}{\partial {r_{i}}^{2}}+v_{ef}(r)\right\rceil \phi_{i}\left(r\right)=\varepsilon_{i}\phi_{i}\left(r\right)$$

where $v_{eff}(r)$ is an effective potential, $\phi_{i}\left(r\right)$ is the Kohn–Sham state, and $\varepsilon_{i}$ is the Kohn–Sham energy eigenvalue. The electron density is given by the following equation:

(22) $$\rho (r)=\sum_{i=1}{\left|\phi_{i}\left(r\right)\right|}^{2}$$

**Figure 10:**
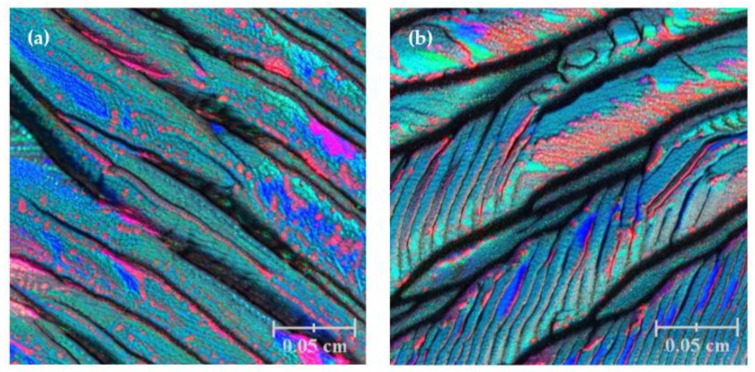
Surface topology on different locations (**a**,**b**) of chromium oxide film.

The thin films are characterized by their surfaces, which can significantly influence their properties. To accurately model the thin-film surface, various substrate treatments are applied. These treatments may involve the use of the vacuum region to stimulate an isolated film, the addition of the vacuum step to mimic the semi-infinite film, or the introduction of appropriate surface terminations [56]. In many cases, a supercell approach is employed to model the thin film. This approach consists of periodically repeating the thin film structured in two dimensions while treating the third dimension as finite. It allows for the investigation of the film properties while minimizing interaction between adjusted films [56] (Figure 10).

Within the domain of exploring magnetic films and their intricate characteristics, DFT simulations have emerged as a predominant computational instrument [56]. DFT presents a quantum-mechanical avenue for comprehending the electronic arrangement and energy aspects of substances, rendering it apt for deciphering the intricate magnetic properties demonstrated by slender films. Nevertheless, akin to any modeling methodology, DFT computations encompass a unique array of strengths and limitations when employed in scrutinizing magnetic films [57].

DFT provides a robust framework for accurately calculating the electronic structure of materials, including magnetic films [57]. It can predict properties such as band structure, density of states, and magnetic moments, offering insights into the underlying physics governing magnetic interactions [58]. DFT can accurately capture magnetic interactions by accounting for the arrangement of electrons’ spins. This enables the study of spin orientations, exchange interactions, and magnetic anisotropy, shedding light on the fundamental mechanisms driving magnetic behavior in films [58]. DFT is versatile and applicable to a wide range of materials, from simple metals to complex compounds. It can be employed to explore the magnetic properties of various compositions, crystal structures, and film thicknesses, aiding in the design and optimization of novel magnetic films [59]. DFT calculations allow for predictive modeling of magnetic films, enabling researchers to design materials with specific magnetic properties. This accelerates material discovery and innovation in fields like data storage, sensors, and spintronics [60]. DFT captures quantum mechanical effects, such as electron tunneling and wave-like behavior, which are vital in understanding and manipulating magnetic behavior at the nanoscale. These effects are pertinent to cutting-edge technologies like quantum computing and nanomagnetic devices [60].

DFT calculations can be computationally demanding, especially for large systems or extended time scales [61]. The need for substantial computational resources limits the size of systems that can be studied and the duration of simulations. DFT calculations rely on exchange-correlation functionals, which are approximations to the complex electron–electron interactions. The choice of functionality can influence the accuracy of results, particularly in strongly correlated systems common in magnetic materials. Modeling magnetic films often requires accurate treatment of surface and interface effects, which can be challenging within DFT [61]. These regions may exhibit different electronic structures and magnetic behaviors compared to the bulk, requiring advanced techniques. DFT calculations are typically performed at absolute zero temperatures and do not inherently capture thermal effects or dynamic processes. Incorporating temperature and dynamics often necessitates additional methodologies and simulations. Interpreting DFT results requires a deep understanding of both quantum mechanics and material-specific considerations. Extracting meaningful physical insights from electronic structure data can be complex, particularly for researchers without a strong background in quantum physics [61] (Table 3).

**Figure 11:**
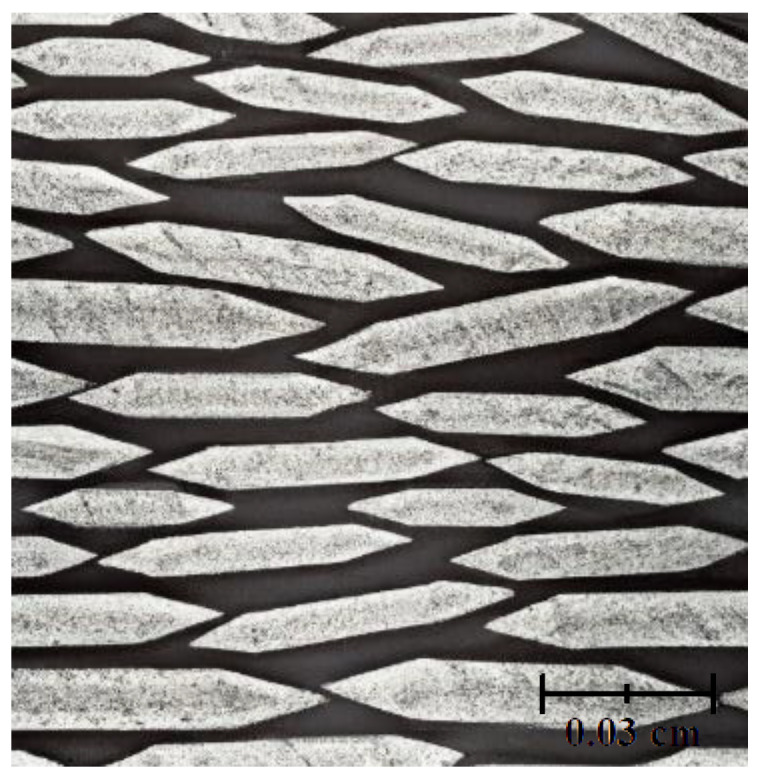
Deposited magnetic film with BaSO_4_ particles.

DFT calculations can also be extended to study the absorption and diffusion of atoms or molecules on the thin-film surface [62] (Figure 11). By calculating absorption energies and diffusion barriers, valuable insight into the reactivity and catalytic properties of the film can be gained. Vibrational properties of thin films can be analyzed using density functional perturbation theory (DFPT) or finite difference methods. These techniques provide information about the photon dispersion, vibrational frequencies, and thermal properties of thin films [62].

In a study related to the DFT and DFPT theoretical investigations of interfacial properties in CaVO_3_ thin films, Beck and Ederer (2020) utilized DFPT and DFT to simulate the impact of the polar CaVO_3_ interface on the physical properties [62] (Figure 11). The results indicate that comparison between experimental and computational results necessitates meticulous attention to the corresponding boundary conditions. In another study conducted by Kaviani and Aschauer (2022), they used density function theory to simulate oxygen vacancies in the system SrMnO_3_, which is grown on SrTiO_3_ substrates. The results highlight that surface and interface effects have a substantial impact on the stability and electric substructure of oxygen vacancies [63].

The next study conducted by Unal et al. (2007) is related to the density functional theory investigation of the initial biolayer grown of Ag on NiAl thin films. Density function theory analysis of supported Ag films on NiAl with an ideal structure reveals that the bilayer growth mode is facilitated by a quantum side effect [64].

After obtaining the results from DFT calculations, post-processing analysis techniques are applied to interpret and analyze the data. This may involve visualizing the charge difference distribution, plotting the band structure, calculating surface energies, or investigating the electronic density of states.

### 3.4. Micromagnetic Simulations

Micromagnetic simulations are a powerful computational technique used to study the magnetization behavior and magnetic properties of thin films and nanostructures at the mesoscopic scale [65]. These techniques provide insight into the spatial distribution of magnetization, magnetostatic interactions, domain structure, and analytics of magnetic systems. Magnetic stimulations treat the magnetic material as a collection of magnetic moments or spins, typically represented on a discrete grid or mesh [65]. Each spin interacts with its neighboring spins through exchange interaction, which governs the alignment and dynamics of the magnetization [66]. The magnetic model considers various energy contributions, including exchange energy, anisotropy energy, magnetostatic energy, and external magnetic field contributions. Micromagnetic simulation involves solving the equation of motion for the magnetic moments or spin over time. This can be achieved using numerical techniques such as the Landau–Lifshitz–Gilbert equation or its variance (23) [66].

(23) $$\frac{dM}{dt}=-\gamma M\times H_{\left(ef\right)}+\alpha M\times (\frac{dM}{dt})$$

where:

M is the magnetization vector of the material.
$H_{\left(ef\right)}$ is the effective magnetic field experienced by the material.
α is the Gilbert damping parameter that characterizes the dissipation of energy during the magnetization dynamics.
γ is the gyromagnetic ratio, a fundamental constant related to the magnetic properties of the material.

The Landau–Lifshitz–Gilbert equation describes the precession and relaxation of magnetization in response to applied magnetic fields and torques. Numerical integration methods, such as the Runge–Kutta algorithm, are commonly used to solve the Landau–Lifshitz–Gilbert equation and simulate magnetization dynamics [67].

Micromagnetic simulation requires input parameters that describe the material properties and geometry of the thin film. These parameters include exchange stiffness, contact, saturation magnetization, anisotropy constant, external magnetic field, and sample dimensions [67]. Some of these parameters can be obtained from experimental measurements, while others can be estimated from theoretical calculations or empirical data. Boundary conditions define the behavior of the spin at the edges of the simulation domain. Common boundary conditions include periodic boundary conditions, which simulate an infinite system, and fixed boundary conditions, which fix the magnetization direction at the edges. The choice of boundary conditions depends on the specific conditions being studied for the desired behavior of the magnetization [68].

Magnetic simulations generate large amounts of data, including the special distribution of magnetization, energy distributions, and dynamic behavior over time [68]. Visualization techniques such as color mapping or vector field representations are used to visualize the magnetization patterns and domain structures. Analysis tools are employed to extract key parameters, such as domain wall velocity, switching fields, or magnetic barriers, from the simulation results [68].

Magnetic stimulation has been successfully applied to a wide range of magnetic systems, including thin films, nanoparticles, magnetic heterostructures, and pattern magnetic structures [69]. They provide valuable insight into magnetization dynamics, domain wall motion, spin waves, and other magnetic phenomena. By adjusting input parameters and studying different geometries and materials, researchers can explore the effect of variable factors on the magnetic behavior of thin films and nanostructures [69].

Micromagnetic simulations have emerged as a crucial computational instrument for comprehending the intricate phenomena displayed by magnetic films [70]. These simulations offer an intermediate-scale perspective on magnetic systems, revealing the dynamics and interplays of individual magnetic moments within the film. While granting profound understandings of magnetic characteristics and conduct, micromagnetic simulations also bring forth specific pros and cons [70].

The micromagnetic methodology is established through a coarse-grained approach, wherein the atomic structure of the system is averaged out and represented by a collection of micromagnetic blocks [71]. Each micromagnetic block encompasses multiple atoms, with their magnetic moments assumed to align parallel to each other. Consequently, the size of these micromagnetic blocks must be smaller than the length scale associated with characteristic magnetic inhomogeneities in the system. This length scale is denoted as $l=min\left(\delta ,\;l_{ex}\right)\;$, where $\delta =\pi \sqrt{A∕K}$ represents the Bloch domain wall width and $l_{ex}=\sqrt{A∕\mu_{0}M^{2}}$ is the exchange length [71]. Here A, K, and M correspond to the exchange stiffness constant, anisotropy density constant, and spontaneous magnetization, respectively. It is noteworthy that the method encounters limitations near temperatures close to the Curie point, where short-wavelength thermal fluctuations become significant. In such cases, a fully atomic description becomes imperative; however, an alternative approach based on the Landau–Lifshitz–Bloch equation is an intriguing option [71].

Moreover, various types of boundary conditions can be adapted to suit the specific requirements of the system under consideration [72].

The orientation of atomic magnetic moments within a micromagnetic block is characterized by a unit vector S*_i_*, denoted as a block spin (with the subscript *i* indicating the block’s position). The complete magnetic state of the system is fully specified by the set of all block spins, denoted as {S*_i_*}. The magnetic energy of the system comprises the following four terms: *E* = *E_H_* + *E_K_* + *E_X_* + *E_D_* (Equations (24)–(26)), where *E_H_*, *E_K_*, *E_X_*, and *E_D_* represent the Zeeman, anisotropy, exchange, and dipolar contributions to energy, respectively [72].

(24) $$E_{H}=-\mu V_{a}\sum MH\cdot S_{i}$$

Here, $\mu =4\times 10^{-7}\;N/A^{2}$ is the vacuum permeability, *V_a_* = *a*^3^ is the micromagnetic block volume, M*i* is the spontaneous magnetization for block *i*, and H is the external magnetic field [73].

Anisotropy energy (E_K_) is calculated as follows:

(25) $$E_{k}=-V_{a}\sum K{\left((n_{i}\cdot S_{i}\right)}^{2})$$

The anisotropy energy accounts for the preference of magnetic moments to align along their easy axis. K_i_ is the anisotropy constant density of block i, and n_i_ is the unit vector along its easy axis [73].

Exchange energy (E_X_) is calculated as follows:

(26) $$E_{x}=-\Sigma J\frac{S_{i}S_{j}}{2}$$

The exchange energy favors neighboring magnetic moments to be aligned. This is described by the Heisenberg formula [73].

Dipolar energy (E_D_) is calculated as follows:

(27) $$E_{D}=-\frac{\mu }{4\pi }\sum J\frac{M_{i}M_{j}}{{\left|r_{i}{-r}_{j}\right|}^{3}}$$

The dipolar energy corresponds to the magnetostatic interaction between classical dipoles [74]. Micromagnetic simulations require knowledge of parameters such as spontaneous magnetization (*M_i_*), anisotropy constant density (*K_i_*), and exchange couplings (*J_ij_*). These parameters are often obtained from experimental data or, alternatively, can be evaluated from first-principle electronic structure calculations. The latter allows for a multiscale approach, providing insights into the variations of parameters near interfaces. However, such calculations can be computationally expensive, and the accuracy of first-principles methods may have limitations for certain materials [74].

In practical applications, parameters such as *M_i_*, *K_i_*, and *J_ij_* may be estimated from experimental data, and the interlayer exchange coupling (IEC) may be treated as a free parameter or approximated as an average of bulk exchanges [74].

**Figure 12:**
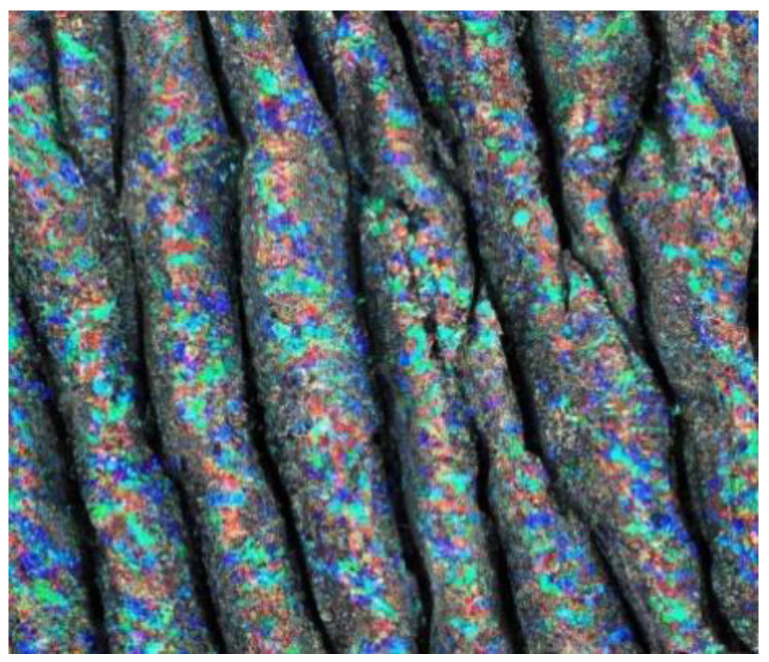
Crystalline chromium oxide film.

Micromagnetic simulations allow researchers to study the behavior of magnetic films with high spatial and temporal resolution [75]. This enables the exploration of dynamic processes such as domain wall motion, magnetization switching, and spin dynamics. Micromagnetic simulations capture the inherent complexity of magnetic systems by incorporating factors like crystal anisotropy, exchange interactions, and external magnetic fields [75]. This realism is essential for accurately representing the behavior of real-world magnetic films. Micromagnetic simulations can handle relatively large systems, encompassing millions of spins, which is crucial for studying realistic magnetic film geometries and sizes. Researchers can systematically investigate the impact of various parameters, such as film thickness, exchange constants, and external fields, on magnetic behavior [75]. This aids in optimizing magnetic films for specific applications. Micromagnetic simulations provide insights into the dynamic evolution of magnetization over time, capturing transient behaviors that are crucial for understanding processes like magnetization precession and relaxation (Figure 12) [75].

Micromagnetic simulations can be computationally demanding, particularly for large systems or when simulating extended time scales [76]. The complexity of the simulations can require substantial computational resources and time. Micromagnetic simulations are often conducted at zero temperature or with minimal consideration of thermal effects. Incorporating finite-temperature effects can be challenging and may require additional modeling approaches. Micromagnetic simulations are classical in nature and do not inherently capture quantum mechanical effects that could be relevant in certain magnetic systems, particularly at the nanoscale [76]. The accuracy of micromagnetic simulations is influenced by the numerical methods and approximations used in the simulation code. The choice of discretization scheme and time-stepping algorithm can impact the fidelity of the results. Extracting meaningful physical insights from micromagnetic simulation data can be challenging, especially for complex systems. Interpretation often requires a deep understanding of both the simulation technique and the underlying magnetic phenomena [76] (Table 4).

An example of successfully applied micromagnetic simulation was a study conducted by Ruiz–Gómez et al. (2023) [77]. In the following study, micromagnetic simulation was extensively used to simulate geometry and magnetic texture. The essential information from the study is that the physics in the film being studied can be accurately captured using micromagnetic simulations with the specified material parameters [77]. There is no need to introduce additional terms or consider the influence of defects. Therefore, micromagnetic simulations are sufficient for carefully predicting the behavior of ultra-thin magnetic nanostructures without the necessity of employing atomistic spin dynamics studies, at least on the scale of several nanometers [77].

Another study is related to the micromagnetic simulation of domains in thin films with different anisotropy directions, which was conducted by Solovev et al. (2020) [78]. The investigation of magnetization processes and domain structures in the films was carried out using micromagnetic simulations. The simulations involve a thin-film model that considers in-plain and perpendicular magnetic anisotropy. The focus of this study was to explore the magnetic microstructure, particularly the equilibrium magnetization configuration and the magnetization processes in the thin films of various thicknesses. The micromagnetic simulation results revealed that when the film thicknesses were equal to slightly larger than a crucial value, a periodic magnetic microstructure formed, characterized by the absence of well-defined domain boundaries [78].

## 4. Challenges and Future Directions

### 4.1. Experimental Validation and Incorporation of Dynamic Effects

Experimental validation of magnetic prediction for films involves conducting experiments to verify the accuracy and variability of theoretical or computational models in predicting the magnetic properties of thin films.

Thin-film samples are prepared with controlled composition, thickness, and structure. Various deposition techniques are employed to deposit thin films onto suitable substrates. Careful control of the position parameter ensures reproducibility and minimizes external influence on the magnetic properties.

Various characterization techniques are employed to measure and analyze the magnetic properties of thin-film samples. These techniques can include methods such as vibrating sample magnetometry (VSM) or superconducting quantum interface device (SQUID) magnetometry, magnetic force microscopy (AFM), magneto-optical measurements (Kerr microscopy), and magnetic resonance techniques (magnetic resonance or nuclear magnetic resonance). The choice of technique depends on specific magnetic properties being investigated, such as magnetization, magnetic anisotropy, coercivity, or magnetic domain structure. Experimental results obtained from the magnetic characterization technique were compared with the predictions made by the theoretical or computational models.

These models may involve fundamental magnetic theories, magnetic simulations, or initial calculations based on the density function theory. The comparison involves evaluating the match between the experimental data and the predicted values for various magnetic parameters, such as magnetic moments, coercive field, or hysteresis behavior. The experimental data in comparison with the characterization are analyzed to identify any discrepancies or deviations. Statistical analysis methods, such as error analysis, regression analysis, or hypothesis testing, may be employed to assess the level of agreement or disagreement between the experimental results and the predicted values. Any observed disparities can provide insights into the limitations and shortcomings of the theoretical models and suggest areas for future improvement. The experimental validation process often involves an interactive approach, where the obtained results and insight from the initial comparison are used to refine the improved theoretical models and suggest areas for further improvement. This iterative process helps to enhance the accuracy and reliability of magnetic prediction and ensures a better understanding of the underlying physical phenomena. Experimental validation of magnetic predictions of thin films is crucial for validating theoretical models, verifying the accessibility of magnetic theories, and providing a basis for further developments in the field.

Incorporation of dynamic effects in the modeling of thin films involves considering the time-dependent behavior and indirections of the magnetic properties and other relevant phenomena within the films. Dynamical effects can be incorporated by performing time-dependent simulations, where the behavior of thin film is studied over a range of time intervals. This involves solving the relevant equations of motion or governing equations using the method that accounts for time evolution. For example, in magnetic thin films, the Landau–Lifshitz–Gilbert equation can be solved to capture the dynamic magnetization behavior, including precision, relaxation, and damping effects. Dynamic effects can be studied by subjecting the thin film to external excitations or perturbations. This can be achieved by applying time-varying magnetic fields, temperature changes, or mechanical strain to the system. The response of the thin film to these excitations can then be analyzed to understand its dynamic behavior. Techniques such as linear response theory, floquet theory, or time-dependent perturbation theory are often employed to characterize the system’s dynamic response. Spin waves and magnons are collective excitations in magnetic materials that play a significant role in their dynamic behavior. Incorporating spin wave, or magnon, and dynamics in the thin-film models allows for the study of wave propagation, dispersion, and interaction phenomena. Techniques such as spin wave theory, micromagnetic stimulations, or dynamic matrix methods can be utilized to incorporate these dynamic effects. Ultrafast laser techniques and time-resolved measurements enabled the study of extremely fast dynamic processes in thin films. By using femtosecond laser pulses and time-resolving detection methods, researchers can prove the ultrafast magnetization dynamics, spin dynamics, and relaxation processes in thin films. These things provide visible insight into processes such as magnetization switching, demagnetization, and ultrafast phase transitions in oil.

Dynamic Monte Carlo simulations are useful for incorporating dynamic effects in the modeling of thin films. Dynamic Monte Carlo simulation captures the stochastic nature of dynamic processes such as surface diffusion, atomic motion, or defect formation. By simulating these processes over time, Dynamic Monte Carlo simulations provide insights into the growth kinetics, surface roughness, or defect evolution in the thin film.

Incorporating dynamic effects in the modeling of thin films enhances the understanding of their time-dependent behavior, transient phenomena, and stability. It provides valuable insight into processes such as magnetization dynamics, spin wave propagation, ultrafast phenomena, and growth kinetics. These dynamic effects are crucial for the design and optimization of thin film-based devices such as magnetic memories, spintronic devices, or sensors (Table 5).

### 4.2. Scaling Up and Integration with Complex Systems

Scaling up complex systems in the modeling of thin films involves extending the study from individual films to multiple interacting films or film–substrate systems. This approach allows researchers to investigate the collective behavior, emergent properties, and interaction between different film layers or between films and substrates. Scaling up complex systems in the modeling of different films required the use of advanced techniques and modern methodologies to capture the intricate relationships and phenomena that arise in such systems.

Many thin-film applications involve the deposition of multiple layers to create complex structures and functional devices. Modeling multilayer systems requires considering the interactions and interfaces between different film layers. This involves incorporating interlayer coupling, such as exchange interactions or the interfacial roughness effect, into the models. Techniques like multilateral simulations, interplay coupling calculations, or effective medium approximations can be employed to study the properties of multilayer thin films. Thin films are often deposited on the substrate, and the interaction between the film and the substrate can have a significant influence on their properties. Modeling the film–substrate interface involves considering factors such as lattice mismatch, strain effect, surface roughness, and interfacial bonding. Techniques such as finite element modeling, lattice mismatch calculation, or atomic simulation can be used to study the effect of film–substrate interface interactions on the structural, mechanical, and electronic properties of the thin films.

Complex systems of thin films can involve different materials with varying properties, such as magnetic films coupled with superconducting or semiconducting layers. The modality of these heterogeneous systems requires the integration of the relevant physics and properties of each material into the models. This can involve incorporating different equations of motion, considering material-specific parameters, or using hybrid modeling techniques that combine different simulation methods. Complex systems on thin films can exhibit coupled phenomena, where the behavior of one film layer influences the properties or behavior of another layer. Examples include exchange coupling between magnetic films, charge transfer between conducting layers, or strain transfer between different films. Modeling coupled phenomena involves considering the interactions, coupling strength, and feedback mechanics between different layers. Techniques such as a couple of differential equations, a couple of field theories, or a couple of simulations can be used to study the dynamics and emergent behavior of these systems.

Integration with our modeling techniques in the modeling of thin films involves combining multiple computational or theoretical approaches to gain a more comprehensive understanding of the film’s behavior and properties.

Atomic modeling techniques, such as molecular dynamics simulation or Monte Carlo simulations, provide detailed information at the atomic scale but are limited in their ability to simulate large systems or long-term scales. To bridge the gap between automatic and continuum scales, atomistic-to-continuum coupling techniques can be employed. These methods aim to connect the automatic model with continuum models, such as finite element analysis (FEA) or continuum mechanics, to simulate the behavior of the thin films over large length and time scales. Multiscale modeling approaches combine models at different length and time scales to capture the hierarchical nature of thin-film systems. This integration involves linking models with different levels of detail, such as automatic, mesoscale, and continuum models. Multiscale methods allow for the study of phenomena that occur across multiple scales, such as defect formation, phase transition, or interface interaction. Techniques, such as concurrent coupling, adaptive mesh refinement, or bridging scale techniques, are employed to ensure consistency and accuracy across different scales.

Integrating the first-principle calculations with other modeling techniques allows for a more comprehensive understanding of the interplay between electrical structure and macroscopic behavior. This integration can involve coupling first-principle calculations with continuum models, embedding DFT calculations with larger simulations, or using DFT-derivate parameters for other models. Integration with experimental data plays a vital role in validating and refining the models. By comparing model predictions with experimental measurements, researchers can access the accuracy and reliability of the models and refine the parameters. This integration involves data simulation techniques, statistical analysis, and optimization algorithms to achieve a better arrangement between the models and experimental data. Additionally, experimental data can be used to inform model development, such as by providing input parameters or boundary conditions.

Hybrid models combine different modeling techniques with a single framework to capture specific aspects of thin-film behavior. For example, combining micromagnetic simulation with finite element analysis allows for the study of magnetization dynamics in the presence of mechanical stress or external fields. Hybrid models can also integrate different physics, such as combining electromagnetic simulation with thermal modeling to study the thermal response of thin films. These methods often involve coupling or exchanging data between different simulations or models.

The Investigation of multiple modeling techniques requires careful consideration of the interfaces, data transfer, and calibration of parameters. Techniques such as data coupling, model calibration, and sensitivity analysis are employed to ensure the consistency and accuracy of the integrated modeling framework. Integrating multiple modeling techniques into thin-film modeling enhances predictive capabilities, widens the scope of the integration, and provides a more comprehensive understanding of film behavior and properties. It allows researchers to study phenomena at different lengths and time scales, capturing complex infrastructures and making more accurate predictions for material design, device optimization, and technological optimization.

## 5. Discussion

In the realm of thin-film systems, a comprehensive inquiry mandates the astute amalgamation of multifarious modeling techniques to engender a holistic understanding of their inherent properties and dynamics. This integrative framework orchestrates the strategic deployment of divergent methodologies to unravel the intricacies enshrined within magnetic films, thereby facilitating advancements across various domains of materials science and applied physics. The ensuing discourse aims to explicate the seamless interplay of these methodological paradigms, thereby affording nuanced insights into the multifaceted landscape of magnetic thin films.

The foundational cornerstone of this investigative endeavor lies in the judicious utilization of DFT calculations. Operative within the quantum mechanical framework, DFT serves as the vanguard for elucidating the electronic structure domain, offering discernment into atomistic configurations, chemical bonding motifs, and intricate electronic interplays manifesting at the interfacial nexus between the thin film and its substrate. These gleanings furnish the epistemic bedrock upon which subsequent explorations into quantum mechanical modeling and micromagnetic simulations are predicated, enabling a granular dissection of specific facets intrinsic to the system’s behavior.

DFT calculations play a pivotal role in providing invaluable insights into the magnetic properties and stability of materials at the atomic scale. Their integration with micromagnetic simulations facilitates the design and optimization of magnetic materials tailored to the exigencies of spintronics and sensing applications. The convergence of these methodologies engenders revolutionary avenues such as the development of skyrmion-based memory devices boasting ultrahigh storage density and minimal power consumption, thereby heralding a paradigm shift in data storage technology. Similarly, the design and implementation of topological insulator-based spintronic devices, underpinned by insights garnered from these computational approaches, hold promise for efficient spin manipulation and transmission, with ramifications spanning spin logic, quantum computing, and spin-based communication realms.

Augmenting this methodological ensemble, the Monte Carlo simulation methodology unfurls a statistical canvas, delineating the intricacies and emergent propensities arising from the stochastic interplay of atomic constituents or magnetic moments within thin-film systems. By elucidating temporal trajectories and stability profiles germane to interfacial dynamics, Monte Carlo simulations provide a comprehensive understanding of the stochastic underpinnings governing magnetic film behavior.

In the domain of thin-film modeling, the discernment of optimal methodologies assumes paramount significance, contingent upon overarching research objectives, attributes earmarked for scrutiny, and the computational resources at one’s disposal. A judicious course of action entails soliciting the insights of domain experts and undertaking a meticulous survey of extant literature. By assimilating the collective wisdom of predecessors and immersing oneself within the rich tapestry of existing knowledge, an informed trajectory for thin-film modeling can be delineated.

As the scholarly odyssey unfolds amidst this intricate tapestry, it becomes imperative to recognize that each method bequeaths a unique facet, converging synergistically towards a comprehensive understanding of magnetic thin-film behavior on substrates. Through the orchestration of a harmonious interplay between DFT calculations, quantum mechanical modeling, micromagnetic simulations, and Monte Carlo simulations, researchers endeavor to unravel the enigmatic labyrinth of magnetic thin-film dynamics, propelling the frontiers of inquiry within spintronics, sensing, and allied disciplines towards unprecedented horizons of scientific exploration and technological innovation.

## 6. Conclusions

In conclusion, the scientific exploration of magnetic films on substrates has unveiled a multifaceted landscape rich with possibilities for advancing technology across various sectors. By delving into the intricacies of magnetic behavior at the interface between thin films and substrates, researchers have gained profound insights into properties such as magnetization coercivity and magnetic anisotropy, critical for optimizing material performance. This endeavor has been bolstered by a suite of advanced modeling techniques, each offering unique perspectives into the underlying physics governing these systems.

As we journey forward, the intricate interplay of modeling techniques promises to shape a future replete with breakthroughs. In this realm of scientific inquiry, where versatility and adaptability reign supreme, the fusion of theoretical insights and computational prowess fuels an unceasing exploration of magnetic thin films, charting pathways to innovation that hold promise for a multitude of applications.

The seamless fusion of these diverse techniques not only enhances our fundamental understanding of magnetic films but also accelerates innovation. The ability to tailor magnetic properties for specific applications, such as flexible electronics and spintronic devices, underscores the practical significance of this endeavor. The symphony of modeling methodologies resonates in unison, propelling our capacity to design novel materials, unravel intricate phenomena, and engineer cutting-edge technologies.

## Figures and Tables

**Table 1:** Advantages and disadvantages of quantum mechanics methods.

Advantages	Disadvantages
-Efficient for large systems, though it oversimplifies details.	-Accuracy may be compromised, especially for complex dynamics.
-Enables parameter study.	-Requires careful parameter tuning for stability.
-Captures realistic behaviors.	-Limited in capturing fine-scale physics.
-Scales well for large systems.	-Interpretation may be complex, requiring validation.
-Compatible with experimental data.	-Simplified models may not capture all dynamics accurately.

**Table 2:** Advantages and disadvantages of Monte Carlo simulations.

Advantages	Disadvantages
-Flexible for complex systems.	-High computational cost for large systems or long simulations.
-Provides accurate results with proper implementation.	-Results are subject to statistical errors, requiring multiple runs for reliability.
-Efficient for certain problems.	-Challenges in accurately modeling complex interactions.
-Allows exploring parameter space.	-May not capture all real-world aspects.
-Incorporates realistic models.	-Outcomes are sensitive to initial conditions.

**Table 3:** Advantages and disadvantages of DFT calculations.

Advantages	Disadvantages
-Efficient for broad applications.	-Relies on approximations, affecting accuracy.
-Applicable to various systems.	-Limited to small-to-medium-sized systems.
-Predicts properties accurately.	-Requires significant computational resources for high accuracy.
-Optimizes atomic structures accurately.	-Sensitive to functional and parameter choices.
-Integrates well with other methods.	-Complex interpretation, especially for non-experts.

**Table 4:** Advantages and disadvantages of micromagnetic simulations.

Advantages	Disadvantages
-Efficient for large systems.	-Oversimplification may lead to inaccuracies.
-Facilitates parameter studies.	-Limited accuracy for complex dynamics.
-Captures realistic behaviors.	-Requires careful tuning for numerical stability.
-Scales well for large systems.	-Limited in capturing fine-scale physics.
-Compatible with experimental data.	-Interpretation may be complex, requiring validation.

**Table 5:** Comparison of simulation methods.

Aspect	Monte Carlo Simulations	DFT	Quantum Mechanics	Micromagnetic Simulations
Description	Utilizes random sampling to obtain numerical results, often applied in statistical mechanics and finance.	Computes the electronic properties of materials by solving the Schrödinger equation by approximating the electron density.	Describes the behavior of particles at the atomic and subatomic levels using mathematical formulations.	Models the behavior of magnetic materials at microscopic scales, simulating magnetic structures and dynamics.
Advancing Features	Efficient for large systems with complex interactions.	Applicable to large systems; used in materials science and chemistry.	Facilitates accurate predictions of molecular structures and reactions.	Valuable for studying magnetic materials and devices in nanotechnology.
Computational Complexity	Typically polynomial, O(N) or O(NlogN).	Cubic complexity is O(N^3^) for traditional methods, but approximations (e.g., LDA, GGA) offer better scalability.	Highly dependent on the specific method used; it can vary from polynomial to exponential.	Typically polynomial, O(N^2^) or O(NlogN) for large-scale simulations.
Additional Features	Allows for the study of systems at equilibrium and non-equilibrium conditions. It enables the simulation of rare events and probabilistic phenomena.	Provides insights into chemical bonding, electronic structures, and the optical properties of materials. Offers accurate predictions of reaction pathways and thermodynamic properties.	It enables the calculation of properties such as energy levels, transition probabilities, and spectroscopic data. Essential for understanding phenomena like quantum tunneling and entanglement.	Allows for the investigation of domain wall dynamics, magnetization reversal processes, and spin transport phenomena. Supports the design and optimization of magnetic memory devices and spintronic components.
Numerical Stability	Highly dependent on sampling scheme and precision; sensitive to statistical errors.	Stability is highly dependent on the choice of exchange-correlation functional and numerical integration techniques.	Numerical stability varies depending on the method; some methods may suffer from convergence issues or numerical instabilities.	Stable numerical schemes exist; numerical stability depends on discretization schemes and time integration methods.
Parallelization	Can be parallelized effectively due to independent sampling; scales well with increasing computational resources.	Parallelization is challenging due to the interdependence of grid points in calculations and the limited scalability of large systems.	Highly parallelizable for certain algorithms and problems; scalability depends on the method and available computational resources.	Parallelization is feasible for large-scale simulations and scales well with the increasing number of computational resources.
Computational Cost	Relatively low computational cost for moderate-precision simulations but can be high for high-precision calculations or rare-event sampling.	Moderate to high computational cost, especially for large systems or high-precision calculations, depends on system size and desired accuracy.	Computational cost varies widely based on the method, system size, and desired accuracy; it can be significant for large systems or high-precision calculations.	Moderate to high computational cost, depending on system size, discretization, and simulation time, can be significant for large-scale simulations with fine spatial or temporal resolutions.
